# Myocardial perfusion is impaired in asymptomatic patients post renal transplantation

**DOI:** 10.1186/1532-429X-15-S1-E68

**Published:** 2013-01-30

**Authors:** Susie Parnham, Suchi Grover, Craig Bradbrook, Govindarajan Srinivasan, Carmine DePasquale, Richard Woodman, Jonathan Gleadle, Joseph Selvanayagam

**Affiliations:** 1 Department of Cardiovascular Medicine, Flinders Medical Centre, Bedford Park, SA, Australia; 2 Renal Medicine, Flinders Medical Centre, Bedford Park, SA, Australia; 3 Flinders Centre for Cardiovascular Magnetic Resonance Research, School of Medicine, Flinders University, Bedford Park, SA, Australia; 4 Flinders Centre for Epidemiology and Biostatistics, School of Medicine, Flinders University, Bedford Park, SA, Australia

## Background

Cardiovascular disease is one of the commonest causes of mortality post-renal transplantation (RT), often in patients with no known cardiac disease. The cardiac phenotype in these patients is not clearly defined. Multi-parametric cardiovascular magnetic resonance (CMR) imaging enables concurrent assessment of myocardial function, perfusion and irreversible injury. We hypothesized that myocardial perfusion reserve would be impaired in asymptomatic post-renal transplant patients when compared with hypertensive controls.

## Methods

Twenty-two asymptomatic RT patients (3 months to 5 years post-transplant) with, no known history of ischemic heart disease) and 12 hypertensive controls underwent CMR scanning at 1.5 T. Myocardial function, late enhancement, and first-pass perfusion at rest and stress was performed. Myocardial Perfusion Reserve Index (MPRI) was calculated as the ratio of hyperemic to resting myocardial blood flow by dividing the myocardial perfusion at stress by rest perfusion (corrected to rate pressure product). All analyses were performed using multivariate linear regression.

## Results

**Figure 1:**
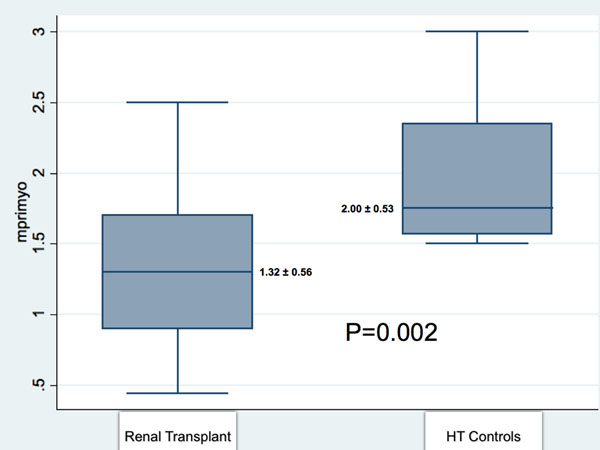
Global myocardial perfusion reserve index (mprimyo)

Baseline clinical characteristics as well as left and right ventricular ejection fraction and volumes were similar for both RT and HT control groups. Mean interventricular septal thickness and LV mass were similar in both renal transplant and hypertensive control groups (LV septum: 1.1 +/- 0.3 cm RT vs 1.1 +/- 0.3 cm HT; LV mass index 127 +/- 35 g/m^2^ RT vs 127 +/- 33 g/m^2^ HT, p>NS) . Rate Pressure Product (RPP) was similar in both groups. Global MPRI was significantly lower in the renal transplant group compared to the hypertensive control group (1.32 ± 0.56 vs 2.00 ± 0.53, P= 0.002, Figure 1). MPRI in all three coronary artery territories were lower in the RT group (see Table 1). One patient in RT group had late Gadolinium enhancement indicating sub-endocardial infarction.

**Table 1:** Myocardial perfusion reserve index in Left Anterior Descending (LAD), Left Circumflex (LCx) and Right Coronary Artery (RCA) territories

Coronary Artery Territories	Renal Transplant MPRI	Hypertensive Control MPRI	P Value
LAD	1.23 ± 0.50	1.93 ± 0.46	0.0005
LCx	1.17 ± 0.53	1.89 ± 0.38	0.0003
RCA	1.24 ± 0.53	1.79 ± 0.31	0.0029

## Conclusions

Asymptomatic patients post renal transplantation have global reductions in myocardial perfusion compared to hypertensive controls, independent of the degree of LVH. This may reflect both severe coronary microvascular disease and/or undiagnosed epicardial coronary artery disease, and may in part explain the poorer cardiac outlook in these patients.

## Funding

Flinders Medical Centre

Flinders Research Centre

